# Advances in Mechanism Research on *Polygonatum* in Prevention and Treatment of Diabetes

**DOI:** 10.3389/fphar.2022.758501

**Published:** 2022-02-08

**Authors:** Shuang Liu, Qiao-Jun Jia, Yi-Qing Peng, Ting-Hui Feng, Shu-Ting Hu, Juan-e Dong, Zong-Suo Liang

**Affiliations:** ^1^ College of Life Sciences, Northwest A & F University, Xi’an, China; ^2^ College of Life Sciences and Medicine, Zhejiang Sci-Tech University, Hangzhou, China

**Keywords:** *Polygonatum*, antidiabetic mechanism, hypoglycemic, hypolipidemic, diabetes

## Abstract

Diabetes mellitus is a fast-growing disease with a major influence on people’s quality of life. Oral hypoglycemic drugs and insulin are currently the main effective drugs in the treatment of diabetes, but chronic consumption of these drugs has certain side effects. Polysaccharides, saponins, flavonoids, and phenolics are the primary secondary metabolites isolated from the rhizomes of *Polygonatum sibiricum* Redouté [Asparagaceae], *Polygonatum kingianum* Collett & Hemsl [Asparagaceae], or *Polygonatum cyrtonema* Hua [Asparagaceae], which have attracted much more attention owing to their unique therapeutic role in the treatment and prevention of diabetes. However, the research on the mechanism of these three *Polygonatum* spp. in diabetes has not been reviewed. This review provides a summary of the research progress of three *Polygonatum* spp. on diabetes and its complications, reveals the potential antidiabetic mechanism of three *Polygonatum* spp., and discusses the effect of different processed products of three *Polygonatum* spp. in treating diabetes, for the sake of a thorough understanding of its effects on the prevention and treatment of diabetes and diabetes complications.

## Introduction

Diabetes mellitus (DM) is a comprehensive endocrine and metabolic disease characterized by glucose metabolism disorders, mainly resulting from insulin resistance or insufficient insulin secretion (Xiao et al., 2019). According to the American Diabetes Association, it is divided into four major types: type 1 diabetes (T1DM), type 2 diabetes (T2DM), gestational diabetes (GDM), and diabetes from other causes. T2DM has the highest incidence among these diseases (Skyler and Oddo, 2010). The key factors that cause T2DM are pancreas β-cell failure, insulin resistance, and its complex interrelationships (Thorens, 2011). More importantly, long-term hyperglycemia may cause malfunction and long-term damage in a variety of tissues and organs, particularly the eyes, nerves, kidneys, heart, and blood vessels (Pham et al., 2019; Sloan, 2019).

In recent years, DM has become one of the primary diseases endangering modern people, and the number of patients has been increasing year by year. Almost all patients require oral hypoglycemic agents or injecting insulin. Diabetes is difficult to control in a maintainable long-term lifestyle (Tahrani et al., 2011; Herman et al., 2018). Current oral hypoglycemic agents include the earlier developed metformin and sulfonamides, as well as some novel hypoglycemic agents targeting the pancreas or liver, such as sodium-dependent glucose transporter 2 (SGLT2) inhibitors, dipeptidyl peptidase-4 (DPP-4) inhibitors, and glucagon-like peptide 1 (GLP-1) receptor agonists, which can enhance insulin activity, exert insulin-like effects, or alleviate glucose metabolism disorders (Tahrani et al., 2016). However, the clinical application of insulin often causes hypoglycemia, insulin resistance, lipoatrophy, and other side effects. Although the newly developed drugs have had a lower risk of hypoglycemia in recent years, they are more expensive than other early-developed drugs such as sulfonylureas, and long-term safety has yet to be determined. Therefore, it is essential to screen the natural products with antidiabetic activity and investigate their material basis, pharmacodynamics, and mechanism of action to provide new ideas for developing high efficiency and low toxicity antidiabetic drugs.

Traditional Chinese medicines (TCMs) are gaining popularity as a result of their success in the treatment and prevention of diabetes, such as *Panax ginseng* C. A. Mey. [Araliaceae], *Lycium barbarum* L. [Solanaceae], *Coptis chinensis* Franch. [Ranunculaceae], *Abelmoschus esculentus* (L.) Moench [Malvaceae], *Angelica sinensis* (Oliv.) Diels [Apiaceae], and *Andrographis paniculata* (Burm.f.) Nees [Acanthaceae] (Xu et al., 2012; Sun et al., 2014; Zhao et al., 2014; Wang et al., 2016a; Cui et al., 2016; Liao et al., 2019). *Polygonatum* is a common genus from the Asparagaceae family widely distributed in China and has been used as medicine or food for more than 2,000 years because it invigorates the spleen, moisturizes the lungs, and invigorates qi. The plant part used is the dry rhizome of *Polygonatum sibiricum* Redouté (*P.sibiricum*), *Polygonatum kingianum* Collett & Hemsl (*P. kingianum*), or *Polygonatum cyrtonema* Hua (*P. cyrtonema*), introduced in the 2020 edition of the *Pharmacopoeia of the People’s Republic of China* (Commission, 2020). The underlying pharmacological applications of *Polygonatum* are gaining popularity in clinical diseases, such as fatty liver disease, Alzheimer’s disease, diabetes mellitus, and cancer (Yu et al., 2021). Such biological activities are closely related to the secondary metabolites of *Polygonatum*, including polysaccharides, saponins, flavonoids, phenolics, alkaloids, anthraquinones, lignans, and a variety of beneficial amino acids (Jiang et al., 2017). Recently, *Polygonatum* spp. have become widely used TCMs in improving diabetes.

In this work, we comprehensively analyzed the antidiabetic-related research work on three *Polygonatum* spp. The purpose of this work is to review the secondary metabolites of *Polygonatum* and their antidiabetic mechanism, investigate the effects of different processed products of *Polygonatum* on treatment, and lay the foundation for the clinical application and product development of *Polygonatum*.

## Data Collection

According to published reports from 2011 to 2021, “*Polygonatum*” or “Rhizoma polygonti” combined with “diabetes” or “anti-diabetic,” “secondary metabolites,” and “processed products” were used as search keywords. The data were collected by various online databases, including PUBMED, Web of Science, Science Direct, SpringerLink, Wiley Online Library, Wanfang, and China Knowledge Network. About 189 papers were found by reading abstracts to exclude repetitive and irrelevant papers. The data were further extracted from the above studies: *P. sibiricum*, *P. kingianum*, and *P. cyrtonema* were used according to the 2020 edition of the *Pharmacopoeia of the People’s Republic of China*; test design with the control group and functional verification; and dose use strictly in line with the standard (rat dose = human dose g * 0.018/0.02 kg). Eventually, we found that 47 articles met the screening standard and brought into this paper by critically reviewing and analyzing the data, aiming to identify secondary metabolites and processed products of *Polygonatum* involved in the antidiabetic mechanism.

## Secondary Metabolites of *Polygonatum*

Currently, the secondary metabolites of *Polygonatum* have been reported to include polysaccharides, saponins (steroidal saponins and triterpenoids), flavonoids, phenols, alkaloids, lignans, phytosterols, and volatile oils, of which the first four ones are the major ingredients and have been studied most frequently. Additionally, polysaccharides and saponins were the highest in *P. cyrtonema*, and flavonoids and other phenolics were the highest in *P. sibiricum* (Table 1).

**TABLE 1:** Comparison of the major chemical constituents of three *Polygonatum* spp.

Species	Polysaccharide (mg/g)	Saponin (mg/g)	Flavonoid (mg/g)	Phenol (mg/g)	References
*Polygonatum sibiricum*	40.68∼123.58	0.289∼2.017	0.018∼0.035	0.013∼0.045	Jiao et al. (2016)
*Polygonatum kingianum*	31.24∼140.94	1.303∼2.845	0.015∼0.030	0.007∼0.029	Jiao et al. (2016)
*Polygonatum cyrtonema*	22.34∼140.94	0.030∼8.920	0.004∼0.034	0.007∼0.038	Jiao et al. (2016)

### Polysaccharides

Polysaccharide is not only an active important component of *Polygonatum* but also an important evaluation index of its quality. It has been reported that polysaccharide is composed of many monosaccharides including fructose (Fru), glucose (Glc), mannose (Man), galactose (Gal), arabinose (Ara), and rhamnose (Rha), as well as a handful of glucuronic acid (GlcA) and xylose (Xyl). The molecular weights of polysaccharides from *Polygonatum* plants are estimated to be approximately 2,734∼3.6 × 10^5^ Da (Zhao et al., 2018). Two new polysaccharides (PSP50-2-1 and PSP50-2-2) were isolated and purified from the rhizome of *P. sibiricum*, both of which were homogeneous polysaccharides by the analysis of the specific optical rotation. Meanwhile, the result of monosaccharide composition indicated that PSP50-2-1 and PSP50-2-2 were made up of Glc, Gal, and Fru (Liu et al., 2021), with the molecular weight of 7.7 and 7.0 kDa, respectively. More importantly, Wang et al. found that four polysaccharides isolated from *P. sibiricum* (PSP1, PSP2, PSP3, and PSP4) were made up of Gal, Rha, Man, Glu, and Xyl in different proportions, and the immune activity of polysaccharides was closely related to that of Rha residues, with the molecular weight of 4.415, 2.236, 7.743, and 6.467 kDa, respectively (Wang et al., 2020). Zhao et al. found that polysaccharides isolated from *P. sibiricum*, *P. kingianum*, and *P. cyrtonema* were mainly made up of Fru and pectins, with a molecular weight of more than 4.1 × 10^5^ Da (Zhao et al., 2020).

### Saponins

Although saponins are another main active component of *Polygonatum*, their content is relatively low. According to the different structures of saponins in *Polygonatum*, saponins were divided into steroidal saponins and triterpenoid saponins. Zhao et al. summarized 162 saponins from 18 species of *Polygonatum* genus, among which 70 steroidal saponins and 12 triterpenoid saponins were isolated from *P. sibiricum*, *P. kingianum*, and *P. cyrtonema* (Zhao et al., 2018)*.* Subsequently, some studies provided novel findings of five novel steroidal saponins isolated from *P. sibiricum*, 3-*O*-β-d-glucopyranosyl(1→2)-β-d-glucopyranosyl(1→4)-β-d-fucopyranosyl-(25*R*)-spirost-5-en-3β,17α-diol, 3-*O*-β-d-glucopyranosyl(1→4)-β-d-fucopyranosyl-(25*R*/*S*)-spirost-5-en-3β,12β-diol, 3-*O*-β-d-glucopyranosyl(1→4)-β-d-fucopyranosyl-(25*R*)-spirost-5-en-3β,17α-diol, 3-*O*-β-d-glucopyranosyl(1→4)-β-d-fucopyranosyl-(25*S*)-spirost-5-en-3β,17α-diol, and kingianoside Z (Zhang et al., 2017; Tang et al., 2019). Two new steroidal saponins were isolated from *P. kingianum*, named polygokingiaside A and polygokingiaside B, respectively (Ha et al., 2021). A novel steroidal saponin was isolated from *P. cyrtonema*, named Huangjingsterol B (Huang et al., 2020). On the other hand, no new triterpenoid saponins were found in *Polygonatum* plants because triterpenoid saponins are found principally in the Magnoliopsida class, and steroidal saponins are distributed widely in the Liliopsida class (Faizal and Geelen, 2013).

### Phenolics

Phenolics include flavonoids, phenolics, and lignins. Flavonoids are ubiquitous in natural plants and have a broad spectrum of biological activities. Until now, 34 flavonoids have been isolated from *P. sibiricum*, *P. kingianum*, and *P. cyrtonema*, which can be divided into six types in accordance with the structure of the parent nucleus: homoisoflavones, isoflavones, flavones, chalcones, dihydroflavones, and rosandalanes (Table 2). Among them, homoisoflavones are the most abundant in *Polygonatum*, such as 4′,5,7-trihydroxy-6-methyl-8-methoxy-homoisoflavanon, disporopsin, and polygonatone H.

**TABLE 2:** Flavonoids isolated from three *Polygonatum* spp.

Number	Name	Source	References
Homoisoflavones			
1	4′,5,7-Trihydroxy-6-methyl-8-methoxy-homoisoflavanon	*Polygonatum sibiricum*	Yu et al. (2016)
2	4′,5,7-Trihydroxy-6-methyl-homoisoflavanon	*P. sibiricum*	Yu et al. (2016)
3	4′,5,7-Trihydroxy-6,8-dimethyl-homoisoflavanon	*P. sibiricum*	Yu et al. (2016)
4	4′,7-Dihydroxy-3′-methoxy-homoisoflavanon	*P. sibiricum*	Yu et al. (2016)
5	2,4,5,7-Tetrallydroxy-homoisoflvanaone	*Polygonatum kingianum*	Jiang et al. (2017)
6	(3R)-5,7-Dihydroxy-8-methyl-3-(2′-hydroxy-4′-methoxybenzyl)-chroman-4-one	*Polygonatum cyrtonema*	Gan et al. (2013)
7	5,7-Dihydroxy-6,8-dimethyl-3-(4′-hydroxybenzyl)-chroman-4-one	*P. cyrtonema*	Wang et al. (2019a)
8	5,7-Dihydroxy-6,8-dimethyl-3-(2′-methoxy-4′-hydroxybenzyl)-chroman-4-one	*P. cyrtonema*	Wang et al. (2019a)
9	5,7-Dihydroxy-6-methyl-3-(4′-hydroxybenzyl)-chroman-4-one	*P. cyrtonema*	Wang et al. (2019a)
10	5,7-Dihydroxy-8-methyl-3-(4′-hydroxybenzyl)-chroman-4-one	*P. cyrtonema*	Wang et al. (2019a)
11	5,7-Dihydroxy-6-methyl-3-(4′-methoxybenzyl)-chroman-4-one	*P. cyrtonema*	Wang et al. (2019a)
12	5,7-Dihydroxy-6,8-dimethyl-3-(4′-methoxybenzyl)-chroman-4-one	*P. cyrtonema*	Wang et al. (2019a)
13	5,7-Dihydroxy-3-(4′-hydroxybenzyl)-chroman-4-one	*P. cyrtonema*	Wang et al. (2019a)
14	5,7-Dihydroxy-6-methyl-3-(2′,4′-dihydroxybenzyl)-chroman-4-one	*P. cyrtonema*	Wang et al. (2019a)
15	5,7-Dihydroxy-3-(2′-hydroxy-4′-methoxybenzyl)-chroman-4-one	*P. cyrtonema*	Wang et al. (2019a)
16	5-Dihydroxy-7-methoxy-6,8-dimethyl-3-(2′-hydroxy-4′-methoxybenzyl)-chroman-4-one	*P. cyrtonema*	Wang et al. (2019a)
17	5,7-Dihydroxy-3-(4′-hydroxybenzylidene)-chroman-4-one	*P. cyrtonema*	Wang et al. (2019a)
18	Disporopsin	*P. cyrtonema*	Wang et al. (2019a)
19	Polygonatone H	*P. cyrtonema*	Wang et al. (2019a)
Isoflavones			
20	Tectoridin	*P. sibiricum*	Jiang et al. (2017)
21	2′,7-Dihydroxy-3′,4′-dimethoxyisoflavanoside	*P. kingianum*	Jiang et al. (2017)
22	2′,7-Dihydroxy-3′,4′-dimethoxyisoflavan	*P. kingianum*	Jiang et al. (2017)
23	4′,7-Dihydroxy-3′-methoxyisoflavone	*P. kingianum*	Jiang et al. (2017)
Chalcones			
24	Isoliquiritigenin	*P. kingianum*	Jiang et al. (2017)
25	Neoisoliquiritigenin	*P. kingianum*	Jiang et al. (2017)
Dihydroflavones			
26	Liquiritin	*P. kingianum*	Jiang et al. (2017)
27	Liquiritigenin	*P. kingianum*	Jiang et al. (2017)
Rosandalanes			
28	Methylnissolin	*P. kingianum*	Jiang et al. (2017)
Flavones			
29	Apigenin-7-glucoside	*P. sibiricum*	Gao et al. (2015)
30	Apigenin-8-*c*-galactoside	*P. sibiricum*	Yu et al. (2016)
31	Kaempferol	*P. sibiricum*	Gao et al. (2015)
32	Myricetin	*P. sibiricum*	Gao et al. (2015)
33	Rutin	*P. sibiricum*	Wang et al. (2016b)
34	Kaempferol-3-*O*-(2″-*O*-β-d-glucopyranosyl)-β-d-glucopyranoside	*P. sibiricum*	Wang et al. (2016b)

Phenolics in plants are secondary metabolites synthesized during the normal development of plants. Relatively rare studies have been conducted on the structural properties of the phenolics and lignans from *Polygonatum*. Wang et al. identified two known compounds (narcissoside and nicotiflorin) from *P. sibiricum* by 1D/2D NMR and MS data (Wang et al., 2016b). Zhai and Wang isolated syringaresinol-di-*O*-β-d-glucoside from *P. sibiricum* (Zhai and Wang, 2018). Chen et al. isolated a benzofuran-type lignan (polygonneolignanoside A) from *P. sibiricum* (Chen et al., 2020).

### Other Secondary Metabolites

The contents of alkaloids, phytosterols, and volatile compounds in *Polygonatum* were extremely low, and their structures were less studied. Polygonatine A and Polygonatine B isolated from *P. sibiricum* were identified as alkaloids (Sun et al., 2005). Four phytosterol compounds have already been identified in *P. sibiricum* and *P. kingianum*, including β-sitosterol, carotenoside, palmitate-3β sitosterol, ester and (22*S*)-cholest-5-ene-1β,3β,16β,22-tetrol 1-*O*-α-l-rhamnopyranosyl 16-*O*-β-d-glucopyranoside (Li et al., 2008; Ahn et al., 2011). Volatile compounds were found in the rhizomes of *P. cyrtonema*, which accounted for 95.97% of the total volatile oils (Yu et al., 2008).

## Potential Antidiabetic Mechanism of *Polygonatum* on Diabetes

Studies have shown that certain active ingredients of traditional Chinese herbal medicines have apparent effects of lowering blood sugar and blood lipids, such as polysaccharides, saponins, flavonoids, phenols, and alkaloids (Xu et al., 2018; Xu et al., 2019; Deng et al., 2020; Hou et al., 2020; Zhuang et al., 2020). *Polygonatum* is rich in these substances and hence is a Chinese herbal medicine with great medicinal value. The number of research papers on secondary metabolites and biological activities of *Polygonatum* is increasing in recent decades.

**FIGURE 1:**
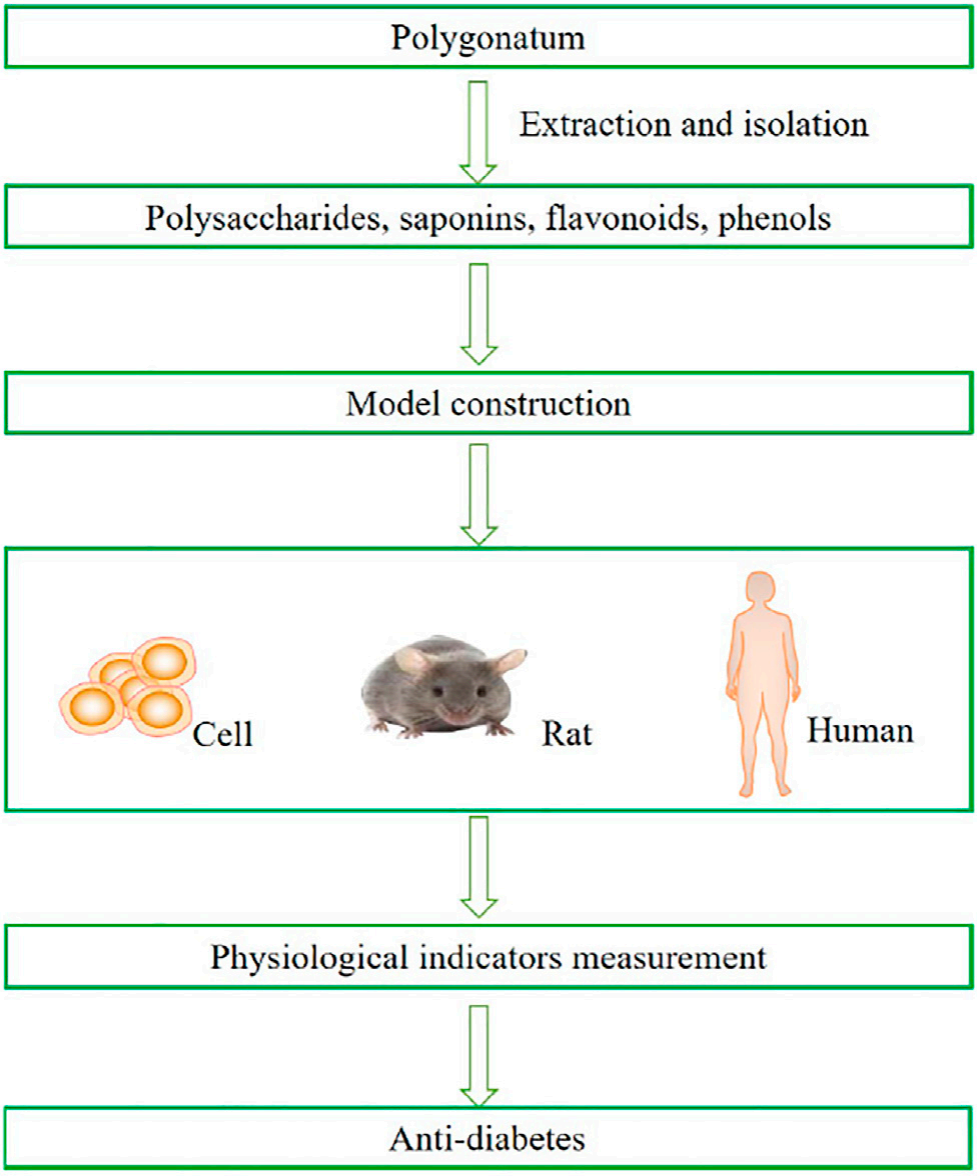
Experimental workflow in the study of *Polygonatum* spp. in the treatment of diabetes.

To date, there are three models to study the antidiabetic mechanism of secondary metabolites from *Polygonatum*: cells, diabetic animal models, and humans (Figure 1). For example, in *in vitro* studies, in which the IR-3T3-L1 adipocytes and IR-HepG2 cells were cultured, it was found that *Polygonatum* could increase glucose intake by alleviating oxidative stress and inflammation (Cai et al., 2019; Luo et al., 2020). In animal models of diabetes, for the sake of identifying the metabolic impact of the *Polygonatum* rhizome extract, high-fat diet (HFD)-, streptozotocin (STZ)-, or alloxan-induced rats were administered *Polygonatum* orally at a certain dose for a period. It is suggested that *Polygonatum* could decrease high blood glucose by analyzing various factors related to metabolic syndrome (Pang et al., 2018; Gu et al., 2020; Li et al., 2020). In addition, *Polygonatum* also improves homeostasis model assessment of insulin sensitivity (HOMA-IS) and homeostasis model assessment of insulin resistance (HOMA-IR) of patients with diabetes in clinical studies (Ping, 2021).

### *In Vitro* Models

The nuclear factor erythroid 2-related factor 2/heme oxygenase-1 (Nrf2/HO-1) signaling pathway is closely related to pancreatic β-cell injury, obesity, glucose metabolism disorders, and insulin resistance. Polysaccharides of *P. sibiricum* (PSP) (50, 100, and 250 μg/ml) can alleviate IR and proliferation of IR-3T3-L1 adipocytes by activating Nrf2/HO-1 signaling pathway in IR-3T3-L1 adipocytes, they promoted the expression of Nrf2 and HO-1 and lessened the expression levels of inflammatory cytokines [interleukin-1β (IL-1β), interleukin-6 (IL-6), and tumor necrosis factor-α (TNF-α)], and subsequently enhanced glucose intake by stimulating the expression of transporter subtype-4 (GLUT4). When the Nrf2 gene was silenced, the expressions of inflammatory cytokines, HO-1, GLUT4, and glucose intake were elevated, thereby reversing the therapeutic effect of PSP in IR-3T3-L1 adipocytes (Cai et al., 2019).

In IR-HepG2 cells, polysaccharides from *P. kingianum* (PKP) enhanced the levels of glucose utilization efficiency at doses of 6.25, 12.5, and 25 mg/L (Li et al., 2020); saponins from *P. sibiricum* (PSS) could significantly inhibit insulin resistance in a dose-dependent manner in HepG2 cells, but it was noteworthy that when the concentration of PSS was above 500 μg/ml, it affected cell viability. Moreover, PSS also could markedly attenuated the activities of α-glucosidase and α-amylase *in vitro* (Luo et al., 2020).

### Animal Models

Polysaccharides of *P. cyrtonema* (PCP) (450–900 mg/kg) significantly improved the survival rate of STZ-induced T1DM female rats by inhibiting weight loss, suppressing inflammatory cytokine expression in the liver, and increasing insulin receptor substrate (IRS) expression, thereby improving the hepatic immune response (Wang et al., 2019b). More importantly, both low (120 mg/kg) and high (480 mg/kg) doses of PKP improved diabetic symptoms by increasing short-chain fatty acid (SCFA) levels, modulating gut microbiota composition, and reducing inflammation in HFD rats (Gu et al., 2020).

Saponins from *P. kingianum* (TSPK) also have antidiabetic effects. STZ-induced diabetic rats were given TSPK for 8 weeks at 0.025 and 0.1 g/kg, TSPK could alleviate hyperlipidemia and hyperglycemia in diabetic rats, and the genome-wide expression indicated that expression of GLUT4 was significantly upregulated. In contrast, the expression of G6P was downregulated in the insulin signal pathway (Lu et al., 2016). The structure and number of gut microbiota of rats treated with TSPK were significantly changed, so TSPK may prevent T2DM by regulating gut microbiota and the secretion of SCFAs (Yan et al., 2017). Furthermore, PSS can activate hexokinase and then converts glucose to glucose-6-phosphatase (G6P), which promotes glycogen synthesis and ultimately reduces insulin resistance. Interestingly, the number of bacteria changed in the dung of the T2DM rats treated with PSS (1, 1.5, and 2 g/kg), with the result that the number of probiotics increased and the number of harmful bacteria decreased (Luo et al., 2020).

Shu et al. found that total flavonoids of *P. sibiricum* (TFP) have significant hypoglycemic effects on both T1DM and T2DM. Compared to those of the control group, the hypoglycemic effects of 100 and 200 mg/kg of TFP were similar to those of 20 mg/kg of acarbose in STZ-induced T1DM rats. In HFD- and alloxan-induced T2DM rats, 200 mg/kg of TFP had a similar hypoglycemic effect to 15 mg/kg of gliclazide. After 9 days of treatment with 100 and 200 mg/kg of TFP, the fasting blood glucose (FBG) of rats decreased in a dose-dependent manner. Besides, TFP significantly inhibited α-amylase activity in a dose-dependent manner *in vitro* (Shu et al., 2012). Overall, TFP may have multiple beneficial effects on lessening hyperglycemia induced by alloxan, STZ, and HFD in diabetic rats, respectively.

However, there is another class of phenolic compound (syringaresinol-di-*O*-β-d-glucoside (SOG)) isolated from *P. sibiricum* that exerts an antidiabetic effect. Treatment with SOG (25, 50, and 75 mg/kg) facilitated insulin secretion and reduced the levels of lipid metabolism and oxidative stress in the STZ-induced diabetic rats, as well as downregulated the expression of nitrotyrosine (NT) and TGF-β1 in kidneys (Zhai and Wang, 2018). Thus, SOG showed a significant antidiabetic effect by suppressing oxidative stress.

In summary, polysaccharides, saponins, flavonoids, and other phenolics of *Polygonatum* have a prominent role in lowering blood sugar and blood lipids in DM (Table 3). The minimum dose of *Polygonatum* secondary metabolites is 25 mg/kg, and the maximum dose is 2 g/kg.

**TABLE 3:** Antidiabetic properties of three *Polygonatum* spp. in cells and animal models.

Species	Part of plant	Compounds	Concentration	Treatment duration	Model	Index	References
*Polygonatum sibiricum*	Rhizome	Polysaccharide	50, 100, and 250 μg/ml	12, 24, and 48 h	IR-3T3-L1 adipocytes	IL-1β, IL-6, and TNF-α↓; Nrf2 and HO-1↑	Cai et al. (2019)
*Polygonatum kingianum*	Rhizome	Polysaccharide	100 mg/L	24 h	IR-HepG2 cells	IRS1/PI3K/Akt↑	Li et al. (2020)
*P. sibiricum*	Rhizome	Saponin	10, 5, 1, 0.5, and 0.1 mg/ml	36 h	IR-HepG2 cells	Glucose consumption, HK, and PK↑	Luo et al. (2020)
*P. sibiricum*	Rhizome	Polysaccharide	200, 400, and 800 mg/kg	12 weeks	STZ-induced diabetic rats	Bax, EGF, p38, VEGF and TGF-β↓; Bcl-2↑	Wang et al. (2019c)
*P. kingianum*	Rhizome	Polysaccharide	120, 240, and 480 mg/kg	14 weeks	HFD rats	FBG, HDL-C, harmful bacteria↓; TC, TG, LDL-C, FINS, beneficial bacteria↑	Gu et al. (2020)
*P. kingianum*	Rhizome	Polysaccharide	1,190 mg/kg	4 weeks	STZ-induced diabetic rats	FBG, HDL-C↓; TC, TG, LDL-C and TC/HDL-C↑	Li et al. (2020)
*P. kingianum*	Rhizome	Polysaccharide	0.1 g/kg	8 weeks	HFD rats	FBG, harmful bacteria↓; FINS, beneficial bacteria↑	Yan et al. (2017)
*Polygonatum cyrtonema*	Rhizome	Polysaccharide	450 and 900 mg/kg	4 weeks	STZ-induced diabetic rats	IL-6, IL-1β↓; IRS-1↑	Wang et al. (2019b)
*P. sibiricum*	Rhizome	Saponin	1, 1.5, or 2 g/kg	11 weeks	STZ-induced diabetic rats	Water consumption, food intake, blood glucose↓ body weight↑	Luo et al. (2020)
*P. sibiricum*	Rhizome	Saponin	100, 200, and 300 mg/kg	2 weeks	Alloxan-induced diabetic rats	blood glucose↓	Pang et al. (2018)
*P. kingianum*	Rhizome	Saponin	0.025 g/kg and 0.1 mg/kg	8 weeks	STZ-induced diabetic rats	G6P↓; GLUT4, PPAR-γ↑	Lu et al. (2016)
*P. kingianum*	Rhizome	Saponin	0.025 and 0.1 g/kg	8 weeks	HFD rats	FBG, harmful bacteria↓; FINS, beneficial bacteria↑	Yan et al. (2017)
*P. sibiricum*	Rhizome	Flavonoid	50, 100, and 200 mg/kg	10 days	Alloxan-induced diabetic rats	Alpha-amylase↓; insulin↑	Shu et al. (2012)
*P. sibiricum*	Rhizome	Phenolic	25, 50, and 75 mg/kg	8 weeks	STZ-induced diabetic rats	TC, TG, LDL-C, FFA, MDA, SOD, CAT, AST, ALT, ALP, and TGF-β1↓; HDL-C, T-AOC↑	Zhai and Wang (2018)
*P. sibiricum*	Rhizome	Polysaccharide	200, 400, and 800 mg/kg	12 weeks	STZ-induced DR rats	FBG, HbA1c, SOD↓; insulin, C-peptide, MDA ↑	Wang et al. (2017)
*P. sibiricum*	Rhizome	Polysaccharide	0.25, 0.5, and 1 g/kg	2 weeks	GM-induced AKI rats	NGAL, KIM-1, IL-1β, IL-6, TNF-α, and p38 MAPK↓	Han et al. (2020)
*P. sibiricum*	Rhizome	Saponin	35 and 70 mg/kg	16 weeks	STZ-induced DN rats	Urea nitrogen, serum creatinine, Wnt4, β-catenin↓	Jing (2019)

Note. IRS, insulin receptor substrate; HK, hexokinase; PK, pyruvate kinase; PPAR-γ, peroxysome proliferator-activated receptor-gamma; FFA, fatty acid; CAT, catalase; AST, aspartate transaminase; ALT, alanine transaminase; ALP, alkaline phosphatase; T-AOC, total antioxidant capacity.

### Clinical Application

To date, clinical studies verified that a few Chinese patent medicines containing *Polygonatum* have a beneficial effect on diabetes, such as Tangwei capsules, Jinlida granules, Tangmaikang granules, Jiangtangjia tablets, and Qizhi Jiangtang capsules (Tables 4, 5). Jinlida granules could significantly decrease the level of hemoglobin A1c (HbA1c) and fasting plasma glucose (FPG) in the 2-h postprandial blood glucose (2hPG) in the individuals who received Jinlida granules (9 g) compared to the control groups (Lian et al., 2019). Jiangtang Tongmai capsules (1.05 g) combined with glibenclamide can reduce the blood glucose level, improve HOMA-IS and HOMA-IR of patients with T2DM, and reduce the severity of clinical symptoms of T2DM (Ping, 2021). HbA1c and HOMA-IR were significantly decreased after treatment with Jiangtangshu tablets (1.5 g) combined with repaglinide, while GLP-1 and fasting serum insulin (FINS) levels were significantly increased (Li and Li, 2019). After treatment with Qizhi Jiangtang capsules (2.5 g), NO serum content was increased, and endothelin-1 (ET-1), thromboxane B2 (TXB2), blood urea nitrogen (BUN), serum creatinine (SCr) contents were lower than those in the control group, which eventually improved renal microcirculation and dysfunction (Si and Xue, 2021). In conclusion, these Chinese patent medicines could effectively control blood glucose and inhibit insulin resistance without significant adverse effects and could be used as an adjuvant drug for the treatment of T2DM and its complications.

**TABLE 4:** Chinese patent medicines containing *Polygonatum* with hypoglycemic effect in human studies.

Drug name	Dosage/times (g)	Cases	Adverse reactions	Index	References
Jiangtang Tongmai capsule	1.05	60	—	HOMA-IS↑; HOMA-IR↓	Ping (2021)
Tangwei capsule	2.5	80	Nausea and dizziness	FBG, 2hPG, HbA1c↓	Chen and Zhang (2019)
Tangmaikang granule	5	102	No	FPG, 2hPG, HbA1c, TG, TC, LDL-C, IL-6↓; HDL-C↑	Yong et al. (2019)
Qizhi Jiangtang capsule	2.5	80	No	ET-1, TXB2, BUN, SCr↓; NO↑	Si and Xue (2021)
Jinlida granule	9	128	Nausea, rash, and heart palpitations	FPG, 2hPG, HbA1c, TC, TG, LDL-C, IL-6, MDA, HOMA-IR↓; HDL-C, SOD, HOMA-β↑	Fan et al. (2021)
Jiangtangshu tablet	1.5	165	Diarrhea, constipation, and abdominal pain	HbA1c, FBG, HOMA-IR↓; GLP-1 and FINS↑	Li and Li (2019)
Jiangtangjia tablet	1.83	38	—	FBG↓	Fan (2012)

**TABLE 5:** Chinese patent medicine prescription containing *Polygonatum* with hypoglycemic effect (data from db.yaozh.com).

Drug name	Sources of prescription	Prescription
Jiangtang Tongmai capsule	National Chinese patent medicine standard assembly Internal medicine Qi blood body fluid subvolume	*Pseudostellaria heterophylla (Miq.)* Pax [Caryophyllaceae; Pseudostellaria Radix], *Astragalus mongholicus* Bunge [Fabaceae; Astragali mongholici radix], *Polygonatum sibiricum* Redouté [Asparagaceae; Polygonati rhizoma], *Asparagus cochinchinensis* (Lour.) Merr [Asparagaceae; Asparagi radix], *Ophiopogon japonicus* (Thunb.) Ker Gawl [Asparagaceae; Ophiopogonis radix], *Scrophularia ningpoensis* Hemsl [Scrophulariaceae; Scrophulariae radix], *Trichosanthes kirilowii* Maxim [Cucurbitaceae; Trichosanthis radix], *Atractylodes lancea* (Thunb.) DC [Asteraceae; Atractylodis rhizoma], *Anemarrhena asphodeloides* Bunge [Asparagaceae; Anemarrhenae rhizoma], *Pueraria lobata* (Willd.) Ohwi [Fabaceae; Puerariae lobatae radix], *Coptis chinensis* Franch [Ranunculaceae; Coptidis rhizoma], *Salvia miltiorrhiza* Bunge [Lamiaceae; Salviae miltiorrhizae radix et rhizoma], *Leonurus japonicus* Houtt [Lamiaceae; Leonuri herba], *Paeonia veitchii* Lynch [Paeoniaceae; Paeoniae radix rubra], *Hirudo niponica* Whitman [Hirudinidae, Hirudo], *Cyathula officinalis* K.C.Kuan [Amaranthaceae; Cyathulae radix], *Spatholobus suberectus* Dunn [Fabaceae; Spatholobi caulis], *Clematis chinensis* Osbeck [Ranunculaceae; Clematidis radix et rhizoma], *Litchi chinensis* Sonn [Sapindaceae; Litchi semen], *Pheretima aspergillum* (E. Perrier) [Megascolecidae; Pheretima], *Conioselinum anthriscoides* ‘Chuanxiong’ [Apiaceae; Chuanxiong rhizoma],Starch
Tangwei capsule	New drug regularization standards 71	*A.* *mongholicus* Bunge [Fabaceae; Astragali mongholici radix], *Panax quinquefolius* L. [Araliaceae; Panacis quinquefolii radix], *P. sibiricum* Redouté [Asparagaceae; Polygonati rhizoma], *T. kirilowii* Maxim [Cucurbitaceae; Trichosanthis radix], *P. lobata* (Willd.) Ohwi [Fabaceae; Puerariae lobatae radix], *C. chinensis* Franch [Ranunculaceae; Coptidis rhizoma], *S. miltiorrhiza* Bunge [Lamiaceae; Salviae miltiorrhizae radix et rhizoma], Glibenclamide
Tangmai Kang granule	*Pharmacopoeia of the People’s Republic of China* 2020 edition	*A.* *mongholicus* Bunge [Fabaceae; Astragali mongholici radix], *Rehmannia glutinosa* (Gaertn.) DC [Orobanchaceae; Rehmanniae radix], *P. veitchii* Lynch [Paeoniaceae; Paeoniae radix rubra], *S. miltiorrhiza* Bunge [Lamiaceae; Salviae miltiorrhizae radix et rhizoma], *Achyranthes bidentata* Blume [Amaranthaceae; Achyranthis bidentatae radix], *O. japonicus* (Thunb.) Ker Gawl [Asparagaceae; Ophiopogonis radix], *P. lobata* (Willd.) Ohwi [Fabaceae; Puerariae lobatae radix], *C. chinensis* Franch [Ranunculaceae; Coptidis rhizoma], Morus alba L. [Moraceae; Mori folium], *P. sibiricum* Redouté [Asparagaceae; Polygonati rhizoma], *Epimedium brevicornu* Maxim [Berberidaceae; Epimedii folium]
Qizhi Jiangtang capsule	*Pharmacopoeia of the People’s Republic of China* 2020 edition	*A.* *mongholicus* Bunge [Fabaceae; Astragali mongholici radix], *R. glutinosa* (Gaertn.) DC [Orobanchaceae; Rehmanniae radix], *P. sibiricum* Redouté [Asparagaceae; Polygonati rhizoma], *H. niponica* Whitman [Hirudinidae, Hirudo]
Jinlida granule	*Pharmacopoeia of the People’s Republic of China* 2010 edition of the third supplement	*Panax ginseng* C.A.Mey [Araliaceae; Ginseng radix et rhizoma], *P. sibiricum* Redouté [Asparagaceae; Polygonati rhizoma], *A. lancea* (Thunb.) DC [Asteraceae; Atractylodis rhizoma], *Sophora flavescens* Aiton [Fabaceae; Sophorae flavescentis radix], *O. japonicus* (Thunb.) Ker Gawl [Asparagaceae; Ophiopogonis radix], *R. glutinosa* (Gaertn.) DC [Orobanchaceae; Rehmanniae radix], *Reynoutria multiflora* (Thunb.) Moldenke [Polygonaceae; Polygoni multiflori radix], *Cornus officinalis* Siebold & Zucc [Cornaceae; Corni fructus], *Poria cocos* (Schw.) Wolf [Polyporaceae; Poria], *C. chinensis* Franch [Ranunculaceae; Coptidis rhizoma], *A. asphodeloides* Bunge [Asparagaceae; Anemarrhenae rhizoma], *E. brevicornu* Maxim [Berberidaceae; Epimedii folium], *S. miltiorrhiza* Bunge [Lamiaceae; Salviae miltiorrhizae radix et rhizoma], *Pueraria montana* var. *thomsonii *(Benth.) M.R.Almeida [Fabaceae; Puerariae thomsonii radix], *L. chinensis* Sonn. [Sapindaceae; Litchi semen], *Lycium chinense* Mill. [Solanaceae; Lycii cortex]
Jiangtang Shu Tablet	New drug regularization standards volume 88	*P. ginseng* C.A.Mey [Araliaceae; Ginseng radix et rhizoma], *Lycium barbarum* L. [Solanaceae; Lycii fructus], *A.* *mongholicus* Bunge [Fabaceae; Astragali mongholici radix], *Eleutherococcus senticosus* (Rupr. & Maxim.) Maxim [Araliaceae; Eleutherococci senticosi rhizoma], *P. sibiricum* Redouté [Asparagaceae; Polygonati rhizoma], *Alpinia oxyphylla* Miq [Zingiberaceae; Alpiniae oxyphyllae fructus], *Ostrea gigas* Thunberg [Ostreidae; Ostreae Concha], *R. glutinosa* (Gaertn.) DC [Orobanchaceae; Rehmanniae radix], *P. lobata* (Willd.) Ohwi [Fabaceae; Puerariae lobatae radix], *S. miltiorrhiza* Bunge [Lamiaceae; Salviae miltiorrhizae radix et rhizoma], *L. chinensis* Sonn. [Sapindaceae; Litchi semen], *A. asphodeloides* Bunge [Asparagaceae; Anemarrhenae rhizoma], *Gypsum Fibrosum*, *Euryale ferox* Salisb [Nymphaeaceae; Euryales semen], *Dioscorea polystachya* Turcz [Dioscoreaceae; Dioscoreae rhizoma], *S. ningpoensis* Hemsl [Scrophulariaceae; Scrophulariae radix], *Schisandra chinensis* (Turcz.) Baill [Schisandraceae; Chinese magnoliavine fruit], *O. japonicus* (Thunb.) Ker Gawl [Asparagaceae; Ophiopogonis radix], *Lindera aggregata* (Sims) Kosterm [Lauraceae; Linderae radix], *T. kirilowii* Maxim [Cucurbitaceae; Trichosanthis radix], *Citrus aurantium* L. [Rutaceae; Aurantii fructus]
Jiangtang jia Tablet	*Pharmacopoeia of the People’s Republic of China* 2020 edition	*A.* *mongholicus* Bunge [Fabaceae; Astragali mongholici radix], *R. glutinosa* (Gaertn.) DC [Orobanchaceae; Rehmanniae radix], *P. sibiricum* Redouté [Asparagaceae; Polygonati rhizoma], *P. heterophylla* (Miq.) Pax [Caryophyllaceae; Pseudostellaria Radix], *T. kirilowii* Maxim [Cucurbitaceae; Trichosanthis radix]

## Potential Antidiabetic Mechanism of *Polygonatum* on Diabetes Complications

Diabetes can also lead to complications of other diseases, such as acute kidney injury (AKI), diabetic retinopathy (DR), and diabetic nephropathy (DN). The p38 MAPK is the most critical and common signaling pathway in protecting against inflammatory kidney injury (Ahmed and Mohamed, 2018). Gentamicin (GM) can stimulate the secretion of inflammatory cytokines via activation of p38 mitogen-activated protein kinase (MAPK)/activation transcription factor 2 (p38 MAPK/ATF2) pathway, triggering a set of inflammatory cascade reactions that result in kidney injury. However, PSP could markedly decrease the expression levels of neutrophil gelatinase-associated lipocalin (NGAL) and kidney injury molecule-1 (KIM-1), preventing the p38 MAPK/ATF2 pathway to suppress the secretion of inflammatory cytokines in the kidney (Han et al., 2020). As a result, PSP has a potential pharmacotherapy on GM-induced AKI rats.

VEGF is a crucial angiogenic growth factor that facilitates the migration, proliferation, and angiogenesis of vascular endothelial cells (Hu et al., 2015). Moreover, some growth factors can promote retinal cell proliferation, such as transforming growth factor-β (TGF-β), which can contribute to cell proliferation and differentiation and suppress DNA synthesis of vascular endothelial cells (Sharma et al., 2015). Epidermal growth factor (EGF) works on the proliferation of retinal capillary endothelial (Sugimoto et al., 2013). However, the treatment of PSP notably reduced the expression of VEGF, TGF-β, and EGF in the DR retina (Wang et al., 2019c). In STZ-induced DR rats, the expression of apoptotic protein B-cell lymphoma-2 factor (Bcl-2) was enhanced, while the expression of Bcl2-associated X protein (Bax) and p38 was reduced in PSP-treated rats. p38 MAPK is pivotal in the regulation of apoptosis. In addition, PSP can also reduce the activity of the superoxide dismutase (SOD) enzyme and increase the content of malondialdehyde (MDA), thus reducing oxidative stress of DM rats (Wang et al., 2017).

Wnt/β-catenin pathway (Wnt) signaling is involved in pancreas development and islet function (Liu and Habener, 2008; Wang et al., 2015; Palsgaard et al., 2016) and plays a vital role in modulating GLP-1 through regulating the transcription of the proglucagon gene in T2DM (Welzel et al., 2009). Zou et al. proved that Shen’an granules could regulate urinary protein, renal function, and dyslipidemia in DN rats, and such effects are achieved by suppressing the activation of the Wnt/β-catenin signaling pathway (Zou et al., 2016). Furthermore, the hypoglycemic effect of PSS on T2DM was also related to the Wnt/β-catenin signaling pathway. There is evidence that the expression of Wnt4 and β-catenin in the DN model group has been notably enhanced compared with that of the control group. In contrast, the expression of Wnt4 and β-catenin in the high-dose and low-dose PSS groups notably decreased (Jing, 2019). Therefore, PSS can suppress the process of tubulointerstitial fibrosis by blocking the activation of the Wnt/β-catenin signaling pathway and finally plays a vital role in kidney protection.

**FIGURE 2:**
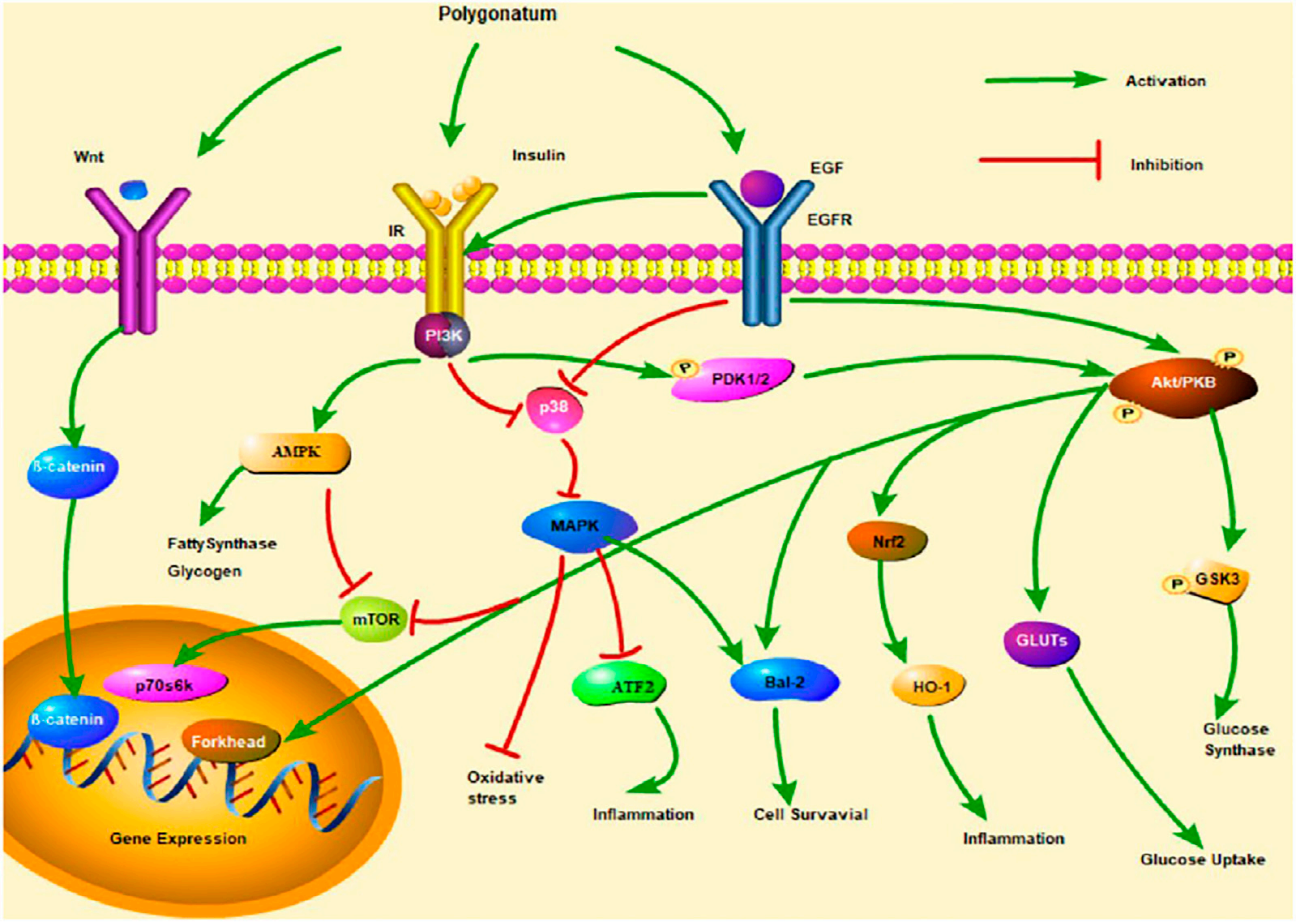
Insulin signaling pathways of three *Polygonatum* spp. Note. mTOR, mammalian target of rapamycin; PDK1/2, 3-phosphoinositide-dependent protein kinase-1/2; GSK3, glycogen synthase kinase 3β.

In brief, the studies of molecular mechanisms suggest that *Polygonatum* influences the development of diabetic complications by regulating MAPK, adenosine monophosphate-activated protein kinase (AMPK), and Wnt/β-catenin signaling pathway (Figure 2).

## Effects of Different Processed Products of *Polygonatum* on Diabetes

TCMs need to be processed to have a better therapeutic effect, unlike Western medicine. Processed TCMs have an apparent therapeutic effect, low toxicity, and convenience for storability. Moreover, different processing methods of the same drug show different efficacy.

An effort was made to compare the effect of hypoglycemic and hypolipidemic among ninefold-processed *P. kingianum* and four products of *P. kingianum* processed with different auxiliary materials (wine, black beans, *Rehmannia glutinosa* (Gaertn.) DC [Orobanchaceae] and *L. barbarum* L. [Solanaceae]). All five processed *P. kingianum* products were administered in high-glucose rats and high-fat rats at doses of 1.95 and 1.35 mg/g, respectively. The results confirmed that the hypoglycemic effect of ninefold-processed *P. kingianum* and *P. kingianum* processed with *R. glutinosa* (Gaertn.) DC. had markedly enhanced effects, while *L. barbarum* L. processed *P. kingianum* shows no significant effect. Additionally, ninefold-processed *P. kingianum* has the best hypolipidemic effect, and *L. barbarum* L. processed *P. kingianum* has the lowest effect through detecting the content of four factors related to lipid metabolism [total cholesterol (TC), triglyceride (TG), high-density lipoprotein cholesterol (HDL-C), and low-density lipoprotein cholesterol (LDL-C)] (Zhang, 2019). Other studies revealed that water extracts from each processed product of *P. sibiricum* (10 g/kg) were given by intragastric administration for 6 weeks; fourfold processing of *P. sibiricum* can better improve Qi and Yin deficiency syndrome by increasing the body weight and tail diameter of rats and regulating the glucose and lipid metabolism, compared to ninefold-processed *P. sibiricum* (Ma et al., 2019).

Li et al. found that fermented *P. sibiricum* (FPS) could lower insulin, FBG, and lipid metabolism than *P. sibiricum*. FPS showed greater efficacy than *P. sibiricum* in decreasing insulin resistance by increasing the p-AKT/AKT ratio, and FPS had a hypolipidemic effect on liver and fat in STZ-induced diabetic rats by improving lipolysis and inhibiting adipogenesis (Li et al., 2021).

## Future Prospects

The beneficial effects of antidiabetes may be related to the metabolites of natural products in the human body. For instance, conjugated (glucuronidated and sulfated) metabolites of hydroxytyrosol and oleuropein are detected in plasma and urine following oleuropein consumption at a single dose of 76.6 mg per person. The concentration of oleuropein metabolites was significantly increased compared with oleuropein (149 vs. 3.55 ng/ml) in plasma (Bock et al., 2013). However, there are no studies on the beneficial effects of chemical components of *Polygonatum* against diabetes, and this may be related to metabolites in humans, and the specific mechanism needs to be further studied. Beyond that, it also is worth further exploring whether different active components of *Polygonatum* work alone or in a particular proportion with better curative effect against diabetes.

## Conclusion

Diabetes mellitus, known as thirst dissipation in ancient China, was characterized by polydipsia, polyuria, polyphagia, emaciation, fatigue, and frequent urination. Now, it is common knowledge that DM is a group of metabolic diseases characterized by hyperglycemia, which is a chronic disease that cannot be cured by pharmaceutical means, but treatments can alleviate the development and symptoms of diabetes. With the increasing number of diabetic patients, natural products of *Polygonatum* (polysaccharides, saponins, flavonoids, and phenols) have attracted wide attention on account of their efficacy in lowering blood sugar and blood lipids. However, there are more studies on the hypoglycemic effect of polysaccharides and saponins than that of flavonoids and phenols. However, flavonoids and other phenolics are worthy of being studied. In addition, this review also summarizes the three insulin signaling pathways—p38MAPK, AMPK, and Wnt/β-catenin signaling pathways—that might be involved in the treatment of diabetes with *Polygonatum*, whereas these signaling pathways could result in a variety of biological activities to change, such as glucose uptake and glycogen synthesis, cell survival, oxidative stress, inflammation, and lipid metabolism. Consequently, the mechanism of action and targets of *Polygonatum* have been studied from the perspective of its unique chemical components, which is crucial to lay the foundation for clinical research.

Preclinical and clinical studies have shown that *Polygonum* has a positive therapeutic effect on diabetes. However, there is still a lack of research on *Polygonum* intake in humans. It is worth noting that the minimum effective dose of *Polygonum* must be determined in clinical studies due to individual differences.

Overall, the antidiabetic efficacy of *Polygonum* is well-known, and the antidiabetic benefits of bioactive components, especially polysaccharides and saponins, have widely been reported. Meanwhile, the combination use of *Polygonatum* and other clinical hypoglycemic drugs could enhance the therapeutic effect of hypoglycemic drugs, giving *Polygonatum* a broader application prospect in the treatment of diabetes and its complications.
